# PHEA-*g*-PMMA Well-Defined Graft Copolymer: ATRP Synthesis, Self-Assembly, and Synchronous Encapsulation of Both Hydrophobic and Hydrophilic Guest Molecules

**DOI:** 10.1038/s41598-017-12710-y

**Published:** 2017-10-03

**Authors:** Aishun Ding, Jie Xu, Guangxin Gu, Guolin Lu, Xiaoyu Huang

**Affiliations:** 1 Department of Materials Science, Fudan University, 220 Handan Road, Shanghai, 200433 People’s Republic of China; 2 Key Laboratory of Synthetic and Self-Assembly Chemistry for Organic Functional Molecules, Shanghai Institute of Organic Chemistry, Chinese Academy of Sciences, 345 Lingling Road, Shanghai, 200032 People’s Republic of China

## Abstract

A series of well-defined amphiphilic graft copolymer bearing a hydrophilic poly(2-hydroxyethyl acrylate) (PHEA) backbone and hydrophobic poly(methyl methacrylate) (PMMA) side chains was synthesized by successive reversible addition-fragmentation chain transfer (RAFT) polymerization and atom transfer radical polymerization (ATRP) through the grafting-from strategy. A well-defined PHEA-based backbone with Cl-containing ATRP initiating group in every repeated unit (*M*_w_/*M*_n_ = 1.08), poly(2-hydroxyethyl 2-((2-chloropropanoyloxy)methyl)acrylate) (PHECPMA), was first prepared by RAFT homopolymerization of 2-hydroxyethyl 2-((2-chloropropanoyloxy)methyl)acrylate (HECPMA), a Cl-containing trifunctional acrylate. ATRP of methyl methacrylate was subsequently initiated by PHECPMA homopolymer to afford the target well-defined poly(2-hydroxyethyl acrylate)-*graft*-poly(methyl methacrylate) (PHEA-*g*-PMMA) graft copolymers (*M*_w_/*M*_n_ ≤ 1.36) with 34 PMMA side chains and 34 pendant hydroxyls in PHEA backbone using CuCl/dHbpy as catalytic system. The critical micelle concentration (*cmc*) of the obtained graft copolymer was determined by fluorescence spectroscopy using *N*-phenyl-1-naphthylamine as probe while micellar morphologies in aqueous media were visualized by transmission electron microscopy. Interestingly, PHEA-*g*-PMMA graft copolymer could self-assemble into large compound micelles rather than common spherical micelles, which can encapsulate hydrophilic rhodamine 6 G and hydrophobic pyrene separately or simultaneously.

## Introduction

The self-assembly of amphiphilic copolymers^1–3^ has gained considerable interests because of its broad potential applications including drug delivery^4^, bioreactor^5^, and catalysis^6^. Researches on the self-assembly of block copolymers revealed that solvent type, ionic strengthen, conditions of micelle preparation, and molecular weight and composition of copolymers all posed significant influences on the aggregated morphology and critical micellar concentration (*cmc*)^7–9^. However, until now most studies focused on linear block copolymers; meanwhile the architecture of copolymers has great impact on their self-assembly properties^10,11^. Nonlinear copolymers would display fundamentally different properties compared to the linear copolymers^12–15^.

Graft copolymer, consisting of backbone and side chains, are endowed with some unusual properties in solution owing to their nonlinear structure^16^. Due to their confined and compact structures, graft copolymer possesses wormlike conformations, compact molecular dimensions, and notable chain end effects; and can form a variety of micellar morphologies in aqueous media^17–23^. Investigation on self-assembly of graft copolymer would deepen the understanding between the structure of copolymer and the morphology in aqueous media, which provides more information about controlling micellar morphologies and designing new nanomaterials^24,25^.

However, studies on graft copolymers have been restricted because the synthesis of well-defined graft copolymers with controlled molecular weights and narrow molecular weight distributions is a much tougher task compared to the synthesis of linear block copolymers. Until now, three common strategies of grafting-through^26,27^, grafting-onto^28,29^, and grafting-from^30,31^ have been widely employed to prepare graft copolymers. The grafting-from strategy, in which polymeric backbone is prepared firstly and the side chains can be attached to the backbone by polymerization of another monomer, becomes the most popular and efficient approach for constructing well-defined graft copolymers with the help of reversible-deactivation radical polymerization (RDRP) including atom transfer radical polymerization (ATRP)^32–34^, reversible addition-fragmentation chain transfer (RAFT) polymerization^35,36^, and single-electron-transfer living radical polymerization (SET-LRP)^37,38^ because RDRP makes the control of molecular weight and molecular weight distribution of side chain become relatively easy.

Recently, much attention has been paid to amphiphilic graft copolymers with hydrophilic backbones and hydrophobic side chains, which could display unique self-assembly morphology^39,40^. In order to achieve the synthesis of this kind of amphiphilic graft copolymers, carboxyls are often introduced into the polymeric chain to maintain its hydrophilicity, of which poly(acrylic acid) (PAA) or poly(methacrylic acid) (PMAA) have been extensively employed to construct the hydrophilic backbones^21,41–44^. Peng *et al*. synthesized an amphiphilic graft copolymer bearing hydrophilic PAA backbone and hydrophobic poly(butyl methacrylate) side chains by a successive two-step ATRP and hydrolysis of a poly(methoxymethyl acrylate) backbone giving a hydrophilic PAA backbone finally^41^. Zhang *et al*. reported the synthesis of PAA-*g*-PMA amphiphilic graft copolymers whose hydrophilic backbones were obtained by selective hydrolysis of P*t*BA-*g*-PMA hydrophobic graft copolymers synthesized via sequential RAFT polymerization and ATRP^42^. However, the conspicuous drawback of the aforementioned work is that the polymeric chain needs to be chemically modified to afford the hydrophilic backbone.

Poly(2-hydroxyethyl acrylate) (PHEA) and poly(2-hydroxyethyl methacrylate) (PHEMA) are also promising to be the hydrophilic backbone of graft copolymers compared to PAA. Nevertheless, PHEA/PHEMA have rarely been employed as hydrophilic backbone because the pendant hydroxyls are often utilized for connecting side chains; for example, the pendant hydroxyls of PHEA/PHEMA can initiate ring opening polymerization (ROP) to afford biodegradable polyester side chains^45^, or be transformed into RDRP initiating groups followed by living/controlled radical polymerization of another monomer^46,47^, or be converted to alkynyls which is able to participate in a click reaction^48^. In 2014, our group developed a new trifunctional acrylate monomer, 2-hydroxyethyl 2-((2-chloropropanoyloxy)methyl)acrylate (HECPMA) comprising a polymerizable double bond, a halogen-containing RDRP initiating group (–OCOCH(CH_3_)Cl), and a hydroxyl simultaneously^19^. The RDRP initiating groups on the backbone are able to initiate ATRP or SET-LRP to introduce the side chains without affecting the pendant hydroxyls^18,19^, which means that amphiphilic graft copolymers with PHEA/PHEMA as the hydrophilic backbone can be obtained directly without the modification of the backbone.

**Figure 1:**
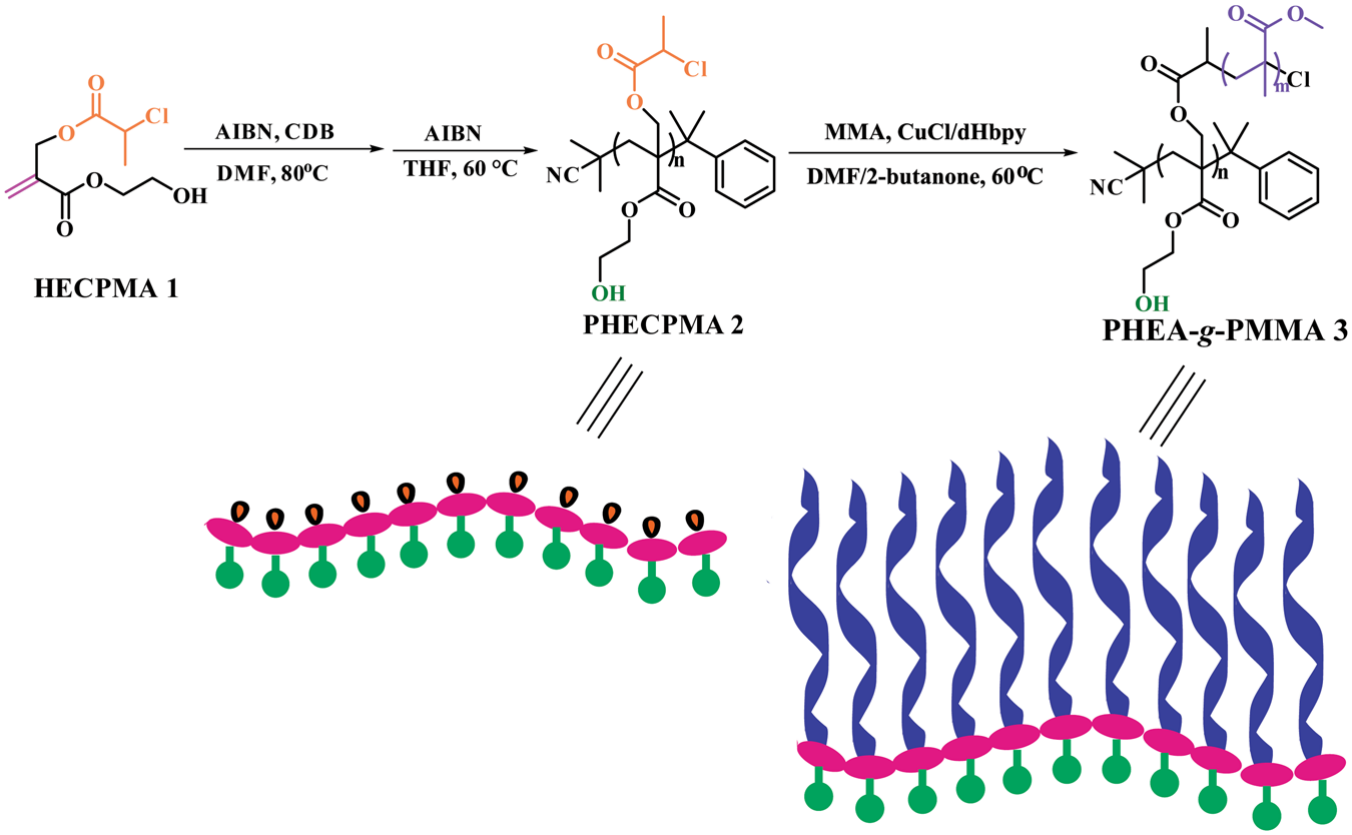
Synthesis of PHEA-*g*-PMMA well-defined amphiphilic graft copolymer.

Herein, for the purpose of expanding the study on amphiphilic graft copolymers bearing PHEA as the hydrophilic backbone, a novel amphiphilic graft copolymer of poly(2-hydroxyethylacrylate)-*graft*-poly(methyl methacrylate) (PHEA-*g*-PMMA) was synthesized by sequential RAFT polymerization and ATRP through the grafting-from strategy as shown in Fig. 1. HECPMA monomer was first polymerized to give a well-defined homopolymer with a pendant ATRP initiating group in every repeated unit followed by ATRP of MMA for affording PHEA-*g*-PMMA. The *cmc* of PHEA-*g*-PMMA amphiphilic graft copolymer in aqueous media was measured by fluorescence spectroscopy and the micellar morphology was preliminarily explored using transmission electron microscopy (TEM).

## Results and Discussion

### Preparation and Characterization of PHECPMA Macroinitiator

MMA, a commonly used methacrylate monomer, has been widely polymerized by RDRP including ATRP, SET-LRP, and RAFT polymerization. Therefore, the most popular grafting-from approach can be employed in the present work to construct the well-defined amphiphilic graft copolymer consisting of hydrophilic PHEA backbone and hydrophobic PMMA side chains through RDRP of MMA initiated by the corresponding pendant initiating groups in the backbone. Since we prefer the well-defined amphiphilic graft copolymer bearing pendant hydroxyls in the backbone, PMMA side chains can not be connected onto the backbone by the usual ester linkage. HECPMA monomer^19^ comprising a polymerizable double bond, a -OCOCH(CH_3_)Cl ATRP initiating group, and a hydroxyl was thus employed as starting material in the current work to construct PHEA-*g*-PMMA well-defined amphiphilic graft copolymer by successive RDRP via the grafting-from strategy.

RAFT homopolymerization of HECPMA monomer was first performed in DMF at 80 °C using AIBN as initiator and CDS as chain transfer agent according to our previous report^19^. The crude product was a kind of pink powder (inset in Figure S1A) because of the residual dithiobenzoate end group originating from CDB and this kind of terminal functionality may influence the following ATRP graft copolymerization of MMA even though its proportion was quite small. Excess AIBN (20 eq.) was then utilized to remove the terminal dithiobenzoate group^42,49^, resulting in a white PHECPMA **2** homopolymer (inset in Figure S1A). UV/vis absorbance spectrum showed the disappearance of the characteristic peak of dithiobenzoate functionality locating at 510 nm, which also illustrated the complete removal of dithiobenzoate end group. The chemical structure of PHECPMA **2** homopolymer was examined by ^1^H NMR, ^13^C NMR (Figure S1), and FT-IR in detail (see Supporting Information).

**Figure 2:**
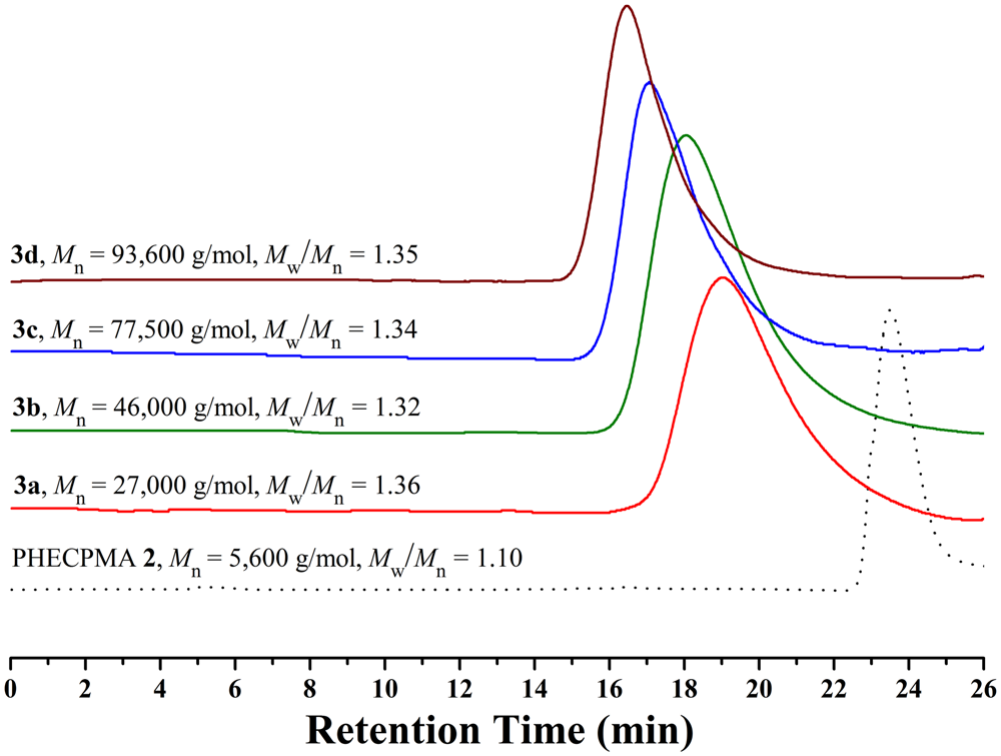
GPC curves of PHECPMA **2** and PHEA-*g*-PMMA **3** in DMF.

GPC curve of PHECPMA **2** homopolymer (Fig. 2) shows a unimodal and symmetric eluent peak with a low polydispersity (*M*_w_/*M*_n_ = 1.10), which demonstrates the well-defined structure of homopolymer **2** prepared by RAFT polymerization of HECPMA monomer. The relative molecular weight of PHECPMA 2 homopolymer is 5,600 g/mol obtained from conventional GPC in DMF. The absolute molecular weight of PHECPMA **2** homopolymer (*M*_n,GPC/MALS_) is determined to be 8,200 g/mol by using GPC/MALS, which is very close to the value (7,900 g/mol) obtained from ^1^H NMR of the homopolymer (Figure S1A). Thus, the number of HECPMA repeated units in the homopolymer was calculated according to eq. 1 (187.3 and 236.6 are the molecular weights of terminal CTA moiety and HECPMA monomer, respectively) and the result is 33.9, which indicates that every PHECPMA 2 chain possesses 33.9 -OCOCH(CH_3_)Cl ATRP initiating groups.

1 $${n}_{{\rm{HECPMA}}}=({M}_{n,\mathrm{GPC}/\mathrm{MALS}}-187.3)/236.6$$

### Synthesis of PHEA-*g*-PMMA Well-Defined Graft Copolymer

ATRP is one of the most useful and powerful RDRP process because of its mild reaction conditions, tolerance of most functional groups, and variety of monomers with precise control over molecular weight and molecular weight distribution. In the current case, the desired PHEA-*g*-PMMA well-defined graft copolymer was constructed by ATRP of MMA via the grafting-from strategy, and the polymerization was initiated by the pendant -OCOCH(CH_3_)Cl initiating groups in PHECPMA **2** homopolymer.

ATRP of MMA was performed in the mixture of DMF/2-butanone (v:v = 1:1) at 60 °C using CuCl/dHbpy as catalytic system. Four PHEA-*g*-PMMA **3** graft copolymers with various molecular weights were obtained by varying the feeding ratio of MMA monomer to the Cl-containing ATRP initiating group (100:1, 200:1, or 600:1) and the polymerization time (20–50 min) as summarized in Table 1. The molecular weights of all four graft copolymers (*M*_n,GPC_ ≥ 27,000 g/mol) are much higher than that of PHECPMA **2** homopolymer (*M*_n,GPC_ = 5,600 g/mol), this indicating the successful ATRP of MMA monomer. GPC curves of four PHEA-*g*-PMMA **3** graft copolymers are shown in Fig. 1 and all four copolymers display a unimodal and symmetric eluent peak with a relatively narrow molecular weight distribution (*M*_w_/*M*_n_ ≤ 1.36), which is also the characteristic of ATRP^32^. In the present work, a high feeding ratio of MMA monomer to ATRP initiating group (more than 100:1) along with a low conversion of monomer (<15%) were conducted in order to suppress the possible intermolecular coupling reaction^50^. Moreover, it can be seen from Table 1 that the molecular weights of copolymer **3** increase with the extending of polymerization time while using the same feeding ratio of MMA to -OCOCH(CH_3_)Cl initiating group (600:1, entry **3c** & **3d**), and with the ascending of the feeding ratio of MMA to -OCOCH(CH_3_)Cl initiating group (100:1 to 200:1, entry **3a** & **3b**) while keeping the polymerization time constant, which are both the characteristic of ATRP^32^.

**Table 1:** Synthesis of PHEA-*g*-PMMA 3 graft copolymer^a^.

Entry	[MMA]:[Cl group]	Time (min)	*M*_n,GPC_^b^ (g/mol)	*M*_w_/*M*_n_^b^	*cmc* ^c^ (g/mL)
**3a**	100:1	50	27,000	1.36	4.12 × 10^−6^
**3b**	200:1	50	46,000	1.32	3.63 × 10^−6^
**3c**	600:1	20	77,500	1.34	2.69 × 10^−6^
**3d**	600:1	40	93,600	1.35	2.03 × 10^−6^

^a^Initiated by PHECPMA 2 macroinitiator (*M*_n,GPC/MALS_ = 8,200 g/mol, *M*_w_/*M*_n_ = 1.08, 33.9 -OCOCH(CH_3_)Cl ATRP initiating groups) in a DMF/2-butanone mixture (v:v = 1:1), [Cl group]:[CuCl]:[dHbpy] = 1:1:1, polymerization temperature: 60 °C. ^b^Measured by GPC in DMF at 35 °C. ^c^Critical micelle concentration determined by fluorescence spectroscopy using PNA as probe.

**Figure 3:**
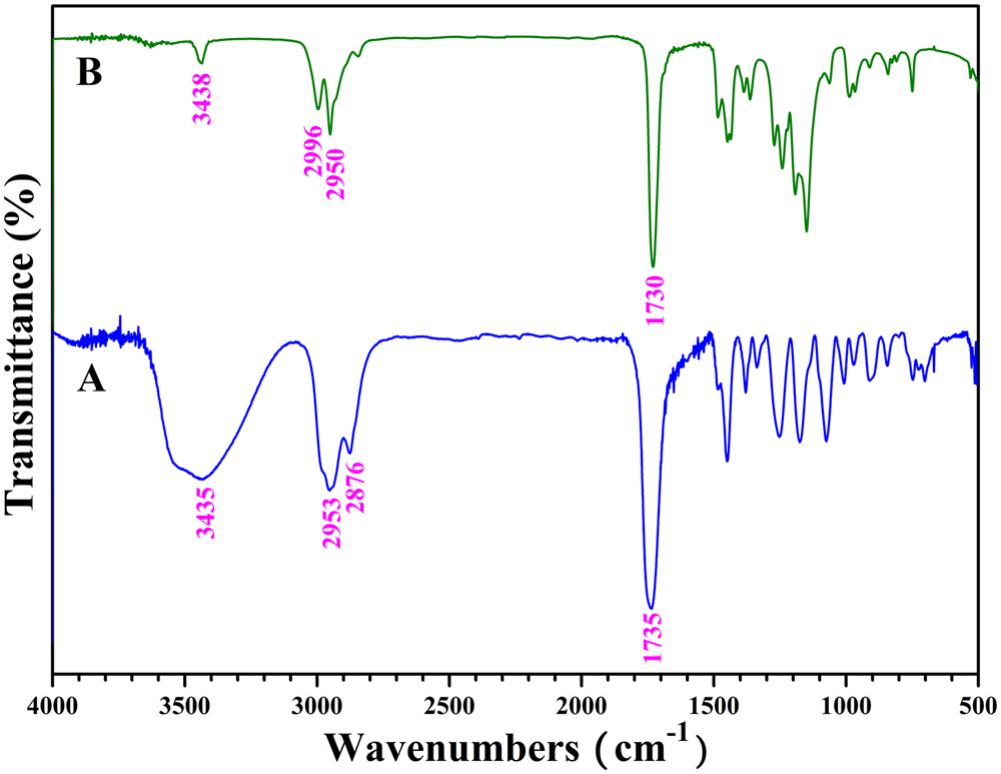
FT-IR spectra of PHECPMA **2** (**A**) and PHEA-*g*-PMMA **3** (**B**).

**Figure 4:**
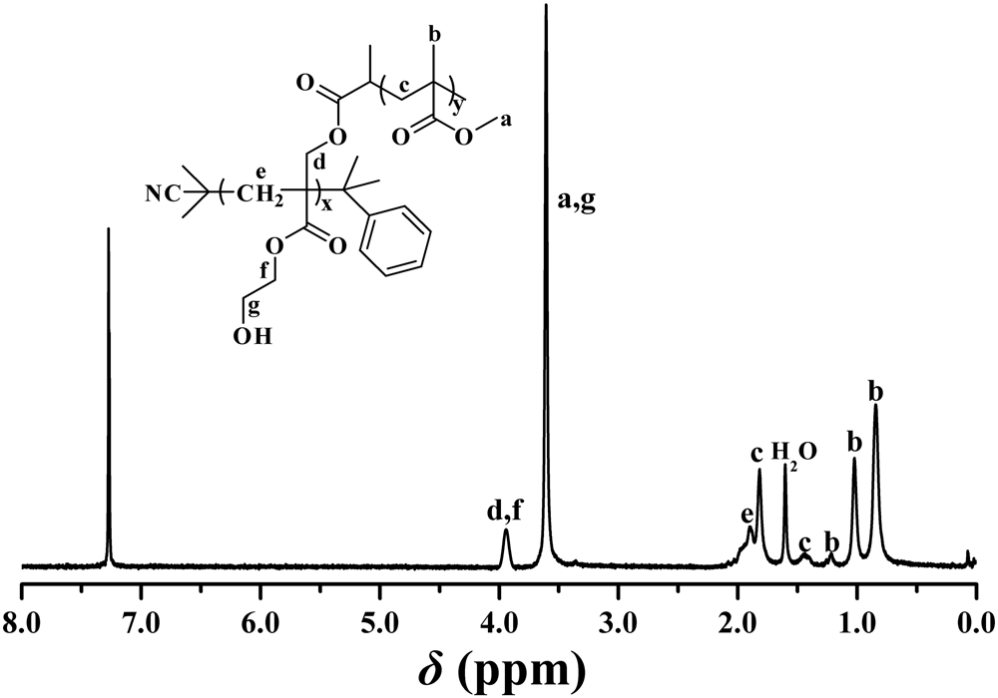
^1^H NMR spectrum of PHEA-*g*-PMMA **3** graft copolymer in CDCl_3_.

FT-IR and ^1^H NMR were utilized to characterize PHEA-*g*-PMMA **3** graft copolymer. In comparison with FT-IR spectrum of PHECPMA **2** macroinitiator (Fig. 3A), the signal of hydroxyl is still observed at 3438 cm^−1^ in FT-IR spectrum after ATRP of MMA as shown in Fig. 2B, however it becomes much weaker because of the introduction of PMMA side chains. Moreover, the sharp peak locating at 1730 cm^−1^ is ascribed to carbonyl existing both in PHEA backbone and PMMA side chains. Figure 4 shows ^1^H NMR spectrum of PHEA-g-PMMA **3** graft copolymer in CDCl_3_ and all corresponding proton resonance signals originating from PHEA backbone and PMMA side chains appear in the spectrum. The characteristic proton resonance signals of PMMA side chains are located at 0.88, 1.03, and 1.22 ppm (peak ‘c’, 3 H, CH_2_C(C*H*_3_)CO_2_). The minor peak at 3.95 ppm belongs to four protons of PHEA backbone (2 H, CO_2_C*H*_2_CH_2_OH and 2 H, CO_2_CC*H*_2_O). In particular, it should be pointed out that we can not find the trace of the proton resonance signal locating at 4.63 ppm attributed to one proton of -OCOC*H*(CH_3_)Cl initiating group (peak ‘b’ in Figure S1A) in Fig. 4, which evidences that the initiation efficiency for ATRP of MMA is 100% in the present work so that we can infer that PHEA-*g*-PMMA **3** graft copolymer possesses 33.9 PMMA side chains. Thus, it is clear that PHEA-*g*-PMMA **3** graft copolymer possesses a well-defined structure: a PHEA backbone (33.9 repeated units) and 33.9 PMMA side chains.

### Self-Assembly Behavior of PHEA-*g*-PMMA Amphiphilic Graft Copolymer

**Figure 5:**
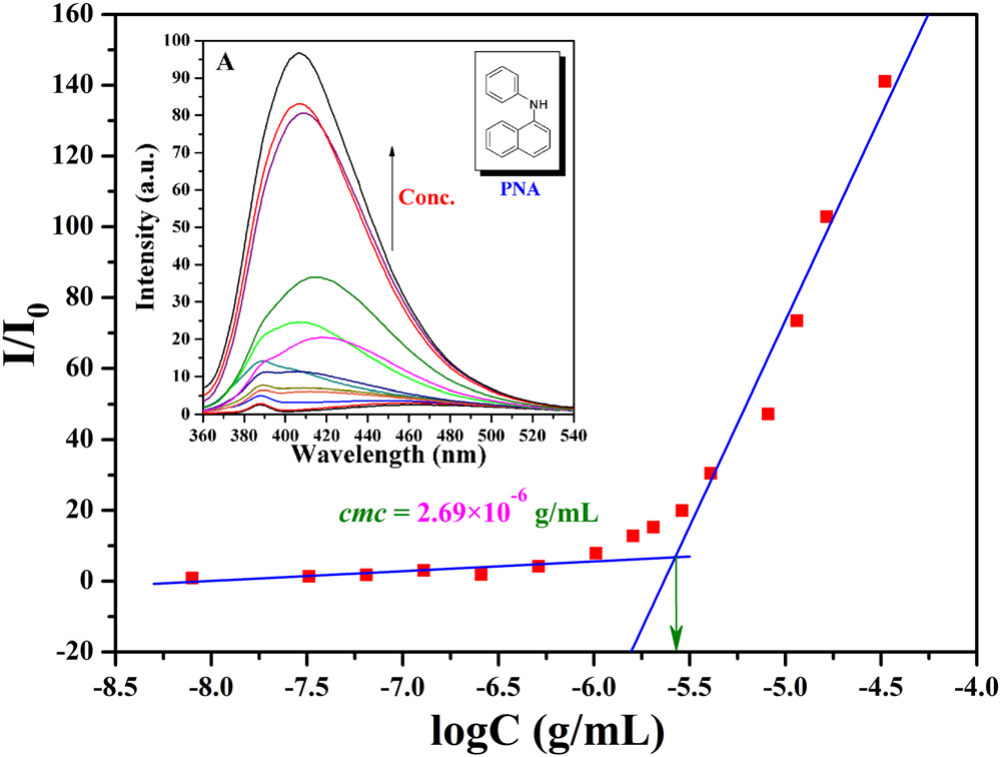
Dependence of fluorescence intensity ratio of PNA emission band at 418 nm ([PNA] = 2 μM) on the concentration of PHEA-*g*-PMMA **3c** at 20 °C.

The amphiphilic nature of PHEA-*g*-PMMA **3** graft copolymer, consisting of hydrophilic PHEA backbone and hydrophobic PMMA side chains, makes the graft copolymer capable of self-assembling in aqueous media so that the graft copolymer possesses a critical micelle concentration (*cmc*) to demonstrate the amphiphilicity. *cmc* of PHEA-*g*-PMMA **3** graft copolymer in aqueous media was determined by fluorescence spectroscopy using PNA as probe. PNA can display higher fluorescence activity in nonpolar surroundings and its fluorescence can be very easily quenched by polar solvents such as water; moreover, it is a more suitable fluorescent probe than pyrene in terms of reproducibility^51^. With the formation of micelles, PNA could be encapsulated into the hydrophobic core of micelles and its fluorescence intensity would increase with the raising of the concentration of polymer. Fluorescence emission spectra of PNA (360–540 nm) in aqueous solution of PHEA-*g*-PMMA **3c** graft copolymer with different concentrations and the relationship of fluorescence intensity ratio (I/I_0_, I and I_0_ refer to the fluorescence intensities of PNA at 418 nm with and without copolymer **3c** in aqueous solution, respectively) of PNA as a function of the concentration of copolymer **3c** are both shown in Fig. 5. It is found that I/I_0_ increases sharply when the concentration of copolymer **3c** exceeds a certain value, indicative of the incorporation of PNA probe into the hydrophobic core of micelles^51^. Thus, *cmc* of PHEA-*g*-PMMA **3c** graft copolymer is determined to be the intersection of two straight lines with a value of 2.69 × 10^−6^ g/mL. As summarized in Table 1, *cmc* values of PHEA-*g*-PMMA **3** graft copolymers are in the range between 4.12 × 10^−6^ g/mL and 2.03 × 10^−6^ g/mL, which are much lower than those of low molecular weight surfactants and are comparable with those of polymeric amphiphiles^52^. Furthermore, one can observe from Table 1 that *cmc* of PHEA-*g*-PMMA **3** graft copolymer falls from 4.12 × 10^−6^ g/mL to 2.03 × 10^−6^ g/mL while increasing the molecular weight of PHEA-*g*-PMMA **3** graft copolymer, i.e. prolonging the length of hydrophobic PMMA side chains with the constant length of hydrophilic PHEA backbone.

**Figure 6:**
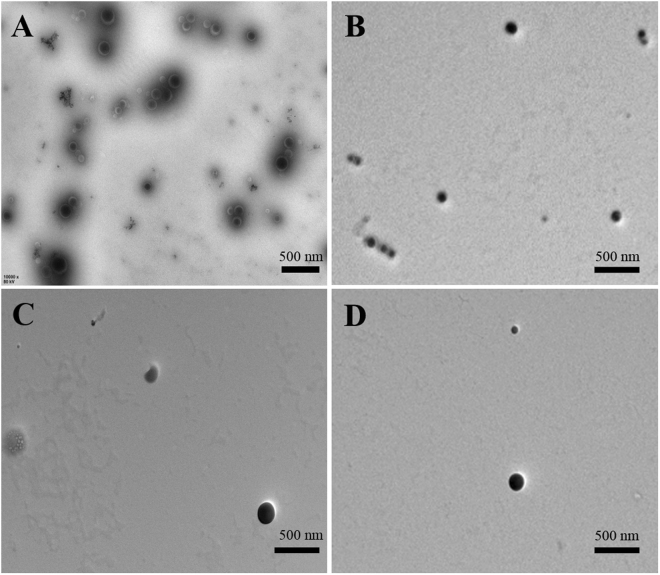
TEM images of micelles formed by PHEA-*g*-PMMA **3a** (**A**), **3b** (**B**), **3c** (**C**), and **3d** (**D**) in aqueous media with a concentration of 1 × 10^−5^ g/mL.

**Figure 7:**
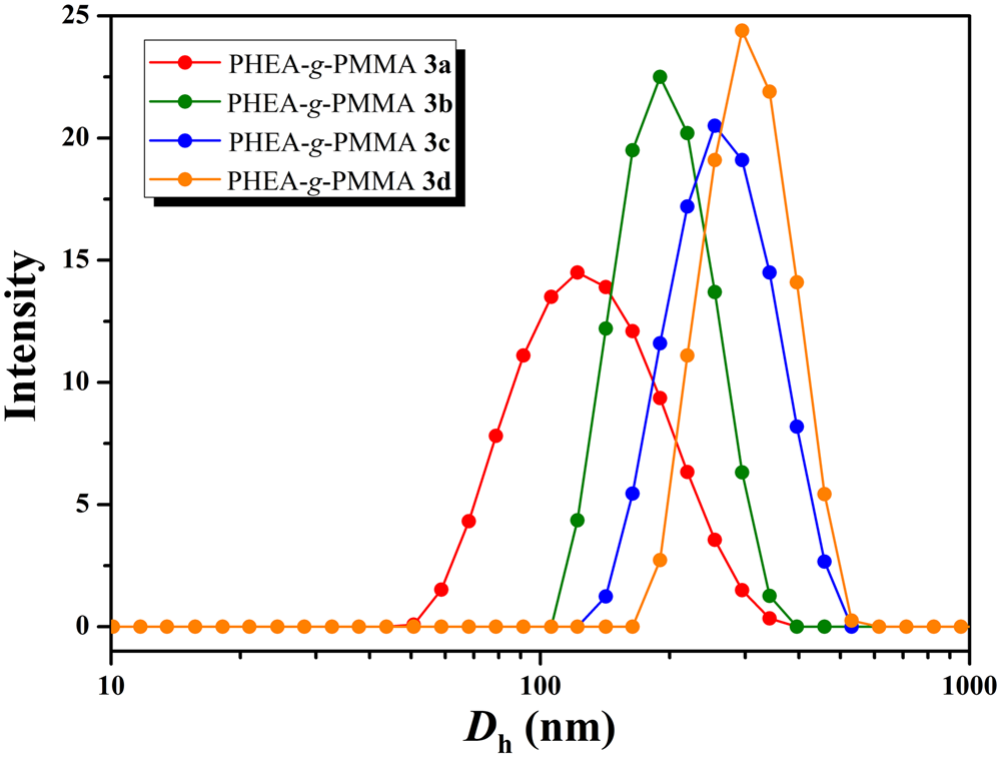
Hydrodynamic diameter distributions of micelles formed by PHEA-*g*- PMMA **3** with a concentration of 1 × 10^−5^ g/mL.

The micellar structures formed by PHEA-*g*-PMMA **3** amphiphilic graft copolymer with a concentration of 0.01 mg/mL in aqueous media (above *cmc*) were visualized by TEM as shown in Fig. 6. All PHEA-g-PMMA **3** graft copolymers with different lengths of PMMA side chains could assemble into spherical micelles. The hydrodynamic diameters (*D*_h_) of these micelles obtained from dynamic light scattering (DLS) are found to be in the range from 122 nm to 294 nm (Fig. 7). However, it should be pointed out that the total lengths of hydrophobic PMMA side chains and hydrophilic PHEA backbone are much shorter than the half of *D*_h_, which signifies that the sphere-like micelles should be large compound micelles, not usual spherical micelles^44,53^. PHEA backbone is considered to form the corona of micelles and the core consists of numerous reverse micelles with PMMA islands in the continuous PHEA phase. It can be seen from Fig. 7 that the size of micelle increases with the ascending of the length of PMMA side chain. We speculate that the size of micelles might be determined by the repulsion between corona chains, i.e. PHEA backbone in the current case. Therefore, the decrease in the content of PHEA segment, i.e. the rising of the length of PMMA side chain, would result in a decrease in repulsion between PHEA chains in the corona of formed micelles, which leads to the increase of the size of micelles.

For the common spherical micelles with a hydrophilic corona and hydrophobic core, only hydrophobic guest molecules can be encapsulated into the hydrophobic domain. Thus, it is a dilemma when both hydrophobic and hydrophilic compounds are required to be encapsulated simultaneously. In this work, as the self-assembly of PHEA-*g*-PMMA **3** graft copolymer in aqueous media is supposed to be large compound micelles with both hydrophilic and hydrophobic domains within the core, it might be able to encapsulate both hydrophobic and hydrophilic agents.

### Encapsulation Capacity of Large Compound Micelles Formed by PHEA-*g*- PMMA Amphiphilic Graft Copolymer

**Figure 8:**
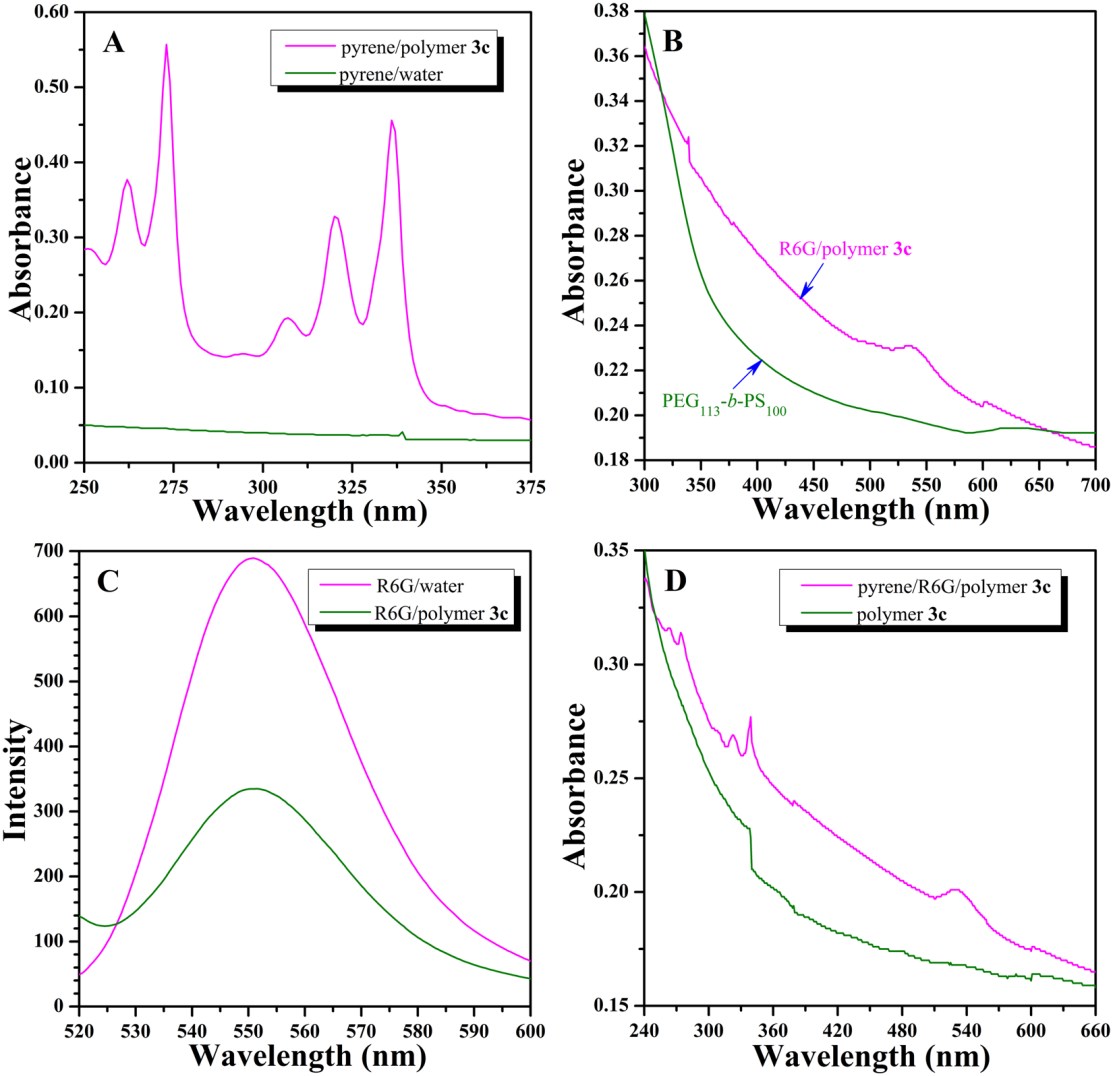
(**A**) UV/vis absorption spectra of pyrene in water and aqueous micellar solution of PHEA-*g*-PMMA **3c**. (**B**) UV/vis absorption spectra of R6G in aqueous micellar solutions of PHEA-*g*-PMMA **3c** and PEG_113_-*b*-PS_100_. (**C**) Fluorescence emission spectra of R6G in water and aqueous micellar solution of PHEA-*g*-PMMA **3c**. (**D**) UV/vis absorption spectra of pyrene and R6G in aqueous micellar solution of PHEA-*g*-PMMA **3c**, and the micellar solution of PHEA-*g*-PMMA **3c** graft copolymer for control experiment.

In order to examine the encapsulation ability of large compound micelles formed by PHEA-*g*-PMMA **3** graft copolymer, pyrene, which is soluble in hydrophobic domain and water-soluble rhodamine 6G (R6G) were chosen as the model guest molecules. Firstly, pyrene was used to test the encapsulation ability of micelles formed by copolymer **3c** (zeta potential: −25.1 mV) for the hydrophobic compound in aqueous media. By using a UV/vis absorption standard curve at 337 nm, the loading content of pyrene is determined to be 26.4 μg pyrene per mg micelle^23^. As pyrene is insoluble in water, almost no UV absorption of pyrene appears in UV/vis absorption spectrum of aqueous solution of pyrene (after filtration to remove the insoluble pyrene) as shown in Fig. 8A (olive line). Nevertheless, it is clear that UV/vis absorption spectrum of aqueous micellar solution of copolymer **3c** (magenta line in Fig. 8A) exhibits a typical UV absorption of pyrene locating at 337 nm, which verifies that pyrene could be captured by micelles to the hydrophobic domains of the core^54–56^.

Next, we used R6G to examine the encapsulation ability of micelles for the hydrophilic compound in aqueous media. The loading content of R6G, 1.2 μg R6G per mg micelle, is also determined by UV/vis spectroscopy using a standard curve at 536 nm^23^. In consideration that the amine group of R6G might be active to react with the pendant Cl at the end of PMMA side chain, therefore we firstly clarified whether R6G is attached to the copolymer by covalent bond. The graft copolymer was recovered from the micellar solution loading with R6G by lyophilization followed by conducting GPC measurement of the obtained graft copolymer using a UV detector (536 nm). The absence of absorption signal at 536 nm (the maximum absorbance for.

R6G) reveals that there is no reaction between amine group of R6G and Cl of graft copolymer under current conditions, i.e. no R6G is covalently linked to the copolymer. Figure 8B (magenta line) shows UV/vis absorption spectrum of R6G-containing aqueous micellar solution of copolymer **3c** (after dialysis for 2 days to remove any free water-soluble R6G) and a characteristic absorption peak of R6G appears at 536 nm. However, turning attention to R6G-containing aqueous micellar solution of PEG_113_-*b*-PS_100_ diblock copolymer (the copolymer aggregates into usual spherical micelles with an average *D*_h_ of 73 nm in aqueous media)^55^, no same peak is detected in UV/vis absorption spectrum (olive line in Fig. 8B). All these evidences mentioned above affirm that hydrophilic R6G could be solubilized within the hydrophilic domains of the core of large compound micelles formed by copolymer **3c** ^57,58^, while the usual spherical micelles formed by PEG_113_-*b*-PS_100_ diblock copolymer fail to capture R6G^23,55^.

It is well-known that fluorescence intensity of R6G in aqueous micellar solution is much lower than that in pure aqueous solution owing to the self-quenching property^55,56^. Moreover, when R6G is dispersed in the aqueous micellar solution, the local concentration of R6G within micelles would be dramatically higher than that of pure aqueous solution of R6G with the same apparent concentration. Therefore, it is possible to confirm whether R6G is really located inside the micelles formed by copolymer **3c** depending on the self-quenching property. The fluorescence intensity of R6G-containing aqueous micellar solution of copolymer **3c** (olive line) is just 48% that of pure aqueous solution of R6G with the same apparent concentration of R6G (magenta line), as shown in Fig. 8C, which demonstrates that R6G in the micellar solution undergoes a self-quenching process. We therefore can infer that R6G is indeed located inside the micelles formed by copolymer **3c**, rather than freely dissolved in water.

Finally, aqueous micellar solution of copolymer **3c** containing both pyrene and R6G simultaneously was prepared. Figure 8D shows two typical UV absorption peaks locating at 337 (pyrene) and 536 (R6G) nm in UV/vis absorption spectrum of R6G/pyrene-containing aqueous micellar solution of copolymer **3c** (magenta line), while they are absent in UV/vis absorption spectrum of aqueous micellar solution of copolymer **3c** (olive line), which illustrates the spontaneous encapsulation of both R6G and pyrene model loading agents within the core of micelles formed by copolymer **3c**. Thus, all aforementioned details distinctly confirm our hypothesis that large compound micelles formed by copolymer **3c** can separately or simultaneously uptake hydrophobic and hydrophilic compounds.

## Conclusions

In the current work, we display the detailed synthesis of PHEA-*g*-PMMA well-defined amphiphilic graft copolymers with relatively narrow molecular weight distributions (*M*_w_/*M*_n_ ≤ 1.36) through sequential RAFT polymerization and ATRP via the most popular grafting-from strategy, using a Cl-containing HECPMA trifunctional monomer as starting material. Post-polymerization functionality transformation is absent during the whole synthesis procedure because HECPMA monomer contains an ATRP initiating group so as to provide a well-defined backbone comprising a certain amount of initiating sites, furthermore affording the target graft copolymers with tunable length of side chains. The self-assembly behavior of PHEA-*g*-PMMA graft copolymer in aqueous media was visualized by TEM, which shows the formation of unusual large compound micelles able to separately or simultaneously uptake hydrophobic pyrene and hydrophilic R6G within the core. The multi-component structure of large compound micelles formed by PHEA-*g*-PMMA graft copolymer has the potential use as a multi-compartment delivery vehicle for the separated or simultaneous uptake of hydrophobic, hydrophilic compounds.

## Electronic supplementary material

Supporting Information
