# Supplementary material for: PHEA-g-PMMA Well-Defined Graft Copolymer: ATRP Synthesis, Self-Assembly, and Synchronous Encapsulation of Both Hydrophobic and Hydrophilic Guest Molecules
Source: Sci Rep. 2017 Oct 3;7:12601. doi: 10.1038/s41598-017-12710-y (PMC5626726; doi:10.1038/s41598-017-12710-y)
Supplement: Supplementary file 1 — Supporting Information [file 41598_2017_12710_MOESM1_ESM.doc]

**Supporting Information for**

**PHEA-*g*-PMMA Well-Defined Graft Copolymer: ATRP Synthesis, Self-Assembly, and Synchronous Encapsulation of Both Hydrophobic and Hydrophilic Guest Molecules**

*Aishun Ding*,*^1^*,*^2^*^.#^ *Jie Xu*, *^1^*^,#^ *Guangxin Gu*, *^1^*,* *Giolin Lu*, *^2^* *Xiaoyu Huang^2^*,***

^1^ Department of Materials Science, Fudan University, 220 Handan Road, Shanghai 200433, People’s Republic of China

^2^ Key Laboratory of Synthetic and Self-Assembly Chemistry for Organic Functional Molecules, Shanghai Institute of Organic Chemistry, Chinese Academy of Sciences, 345 Lingling Road, Shanghai 200032, People’s Republic of China

**Experimental section**

**Materials**

Methyl methacrylate (MMA, Aldrich, 99%) was washed with 5% aqueous NaOH solution to remove the inhibitor, then washed with water, dried over CaCl_2_ and distilled twice *in vacuo* from CaH_2_ prior to use. 2,2’-Azobis(isobutyronitrile) (AIBN, Aldrich, 98%) was recrystallized from anhydrous ethanol twice. Copper (I) chloride (CuCl, Aldrich, 98%) was purified by stirring overnight over CH_3_CO_2_H at room temperature, followed by washing the solid with ethanol, diethyl ether, and acetone prior to drying at 40^o^C *in vacuo* for one day. *N*-Phenyl-1-naphthylamine (PNA, Alfa Aesar, 97%) was purified by recrystallization in ethanol three times. Dimethyl formamide (DMF, Aldrich, 99.8%) was dried over KOH and distilled from CaH_2_ under N_2_ prior to use. 2-Butanone (Aldrich, 99%) was dried over CaSO_4_ and distilled under N_2_ prior to use. Tetrahydrofuran (THF, Aldrich, 99%) was dried over CaH_2_ and distilled from sodium and benzophenone under N_2_ prior to use. Pyrene (Aldrich, 99%) and rhodamine 6G (R6G, Aldrich, 99%) were used as received. 2-Hydroxyethyl 2-((2-chloropropanoyloxy)methyl)acrylate (HECPMA),^1^ cumyl dithiobenzoate (CDB),^2^ and diheptyl-2,2'-bipyridine (dHbpy)^3^ were synthesized according to previous literatures.

**Characterizations**

FT-IR spectra were recorded on a Nicolet AVATAR-360 FT-IR spectrophotometer with a resolution of 4 cm^-1^. All ^1^H (400 MHz) and ^13^C (100 MHz) NMR analyses were performed in DMSO-*d*_6_ and CDCl_3_ on a JEOL JNM-ECZ400 spectrometer. Relative molecular weights and molecular weight distributions were measured by conventional gel permeation chromatography (GPC) system equipped with a Waters 1515 Isocratic HPLC pump, a Waters 2414 refractive index detector, and a set of Waters Styragel columns (HR3 (500-30,000), HR4 (5,000-600,000), and HR5 (50,000-4,000,000), 7.8×300 mm, particle size: 5 μm). GPC measurements were carried out at 35^o^C using LiBr-added DMF ([LiBr] = 15 mM) as eluent (flow rate: 1.0 mL/min). The system was calibrated with linear poly(methyl methacrylate) standards. Absolute molecular weight of the macroinitiator was determined by GPC equipped with a multiangle light scattering detector (GPC/MALS), DMF was used as the eluent with a flow rate of 1.0 mL/min, detectors: Wyatt Optilab rEX refractive index detector and Wyatt DAWN HELEOS 18-angle light scattering detector with a 50 mW solid-state laser operating at 658 nm. UV/vis spectra were measured by a Hitachi U-2910 [spectrophotometer](javascript:popupOBO('CMO:0002232','c2py20933k')) with a rate of 200 nm/min. Steady-state fluorescence spectra were measured at 20^o^C on a Hitachi F-2700 spectrophotometer with the band width of 5 nm for excitation and emission, the emission intensity at 418 nm (λ_ex_ = 340 nm) was recorded to determine the critical micelle concentration (*cmc*) and the emission intensity of R6G at 551 nm (λ_ex_ = 500 nm) was recorded. Hydrodynamic diameter (*D*_h_) and zeta potential were measured by a Malvern Nano-ZS90 Zetasizer. TEM images were obtained by a JEOL JEM-2100 instrument operated at 80 kV.

**RAFT Homopolymerization of HECPMA**

AIBN (9.3 mg, 0.057 mmol) and CDB (46.3 mg, 0.17 mmol) were first added into a 10 mL Schlenk flask (flame-dried under vacuum prior to use) sealed with a rubber septum for degassing and kept under N_2_. Next, HECPMA **1** (1.00 g, 4.25 mmol) and anhydrous DMF (0.22 mL) were added via a gastight syringe. The flask was degassed by three cycles of freezing-pumping-thawing followed by immersing the flask into an oil bath set at 80^o^C. The polymerization was terminated by immersing the flask into liquid N_2_ after 8 h. THF was added for dilution and the solution was precipitated into cold diethyl ether. The crude product was purified by repeated dissolution and precipitation followed by drying *in* *vacuo* overnight to give 0.832 g of pink powder.

To remove the dithiobenzoate end moiety, AIBN (0.46 g, 2.8 mmol) and 0.7 g of pink powder (0.085 mmol of dithiobenzoate group determined from *M*_n,GPC/MALS_ = 8,223 g/mol) were first added to a 100 mL Schlenk flask (flame-dried under vacuum prior to use) sealed with a rubber septum for degassing and kept under N_2_. Next, 30 mL of anhydrous THF was added via a gastight syringe. The flask was immersed into an oil bath set at 60^o^C and the reaction was quenched by liquid N_2_ after 48 h. The solution turned colorless and was precipitated into cold diethyl ether after concentration. After repeated purification via dissolution and precipitation, 0.50 g of white powder, poly(2-hydroxyethyl 2-((2-chloropropanoyloxy)methyl)acrylate) (PHECPMA) **2**, was obtained by drying *in* *vacuo* overnight. GPC: *M*_n,GPC_ = 5,600 g/mol, *M*_w_/*M*_n_ = 1.10. GPC/MALS: *M*_n,GPC/MALS_ = 8,223 g/mol, *M*_w_/*M*_n_ = 1.08. FT-IR: *ν* (cm^-1^): 3433 (*ν*_O-H_), 2951 (*ν*_C-H_), 2878 (*ν*_C-H_), 1735 (*ν*_C=O_), 1450, 1380, 1253, 1174, 1076, 1008, 970, 912, 844, 748. ^1^H NMR (DMSO-*d*_6_): *δ* (ppm): 1.18 (12H, terminal C(C*H*_3_)_2_), 1.66 (3H, CHClC*H*_3_), 1.98 (2H, C*H*_2_CCO_2_), 3.56 (2H, CO_2_CH_2_C*H*_2_OH), 3.82 (2H, CO_2_C*H*_2_CH_2_OH), 4.07 (2H, CO_2_CC*H*_2_O), 4.63 (1H, C*H*ClCH_3_), 4.88 (1H, CH_2_O*H*), 7.11-7.30 (5H, terminal C_6_*H*_5_). ^13^C NMR (DMSO-*d*_6_): *δ* (ppm): 21.8 (CHCl*C*H_3_), 44.3 (*C*H_2_CCO_2_), 47.8 (*C*HClCH_3_), 53.3 (CO_2_CH_2_*C*H_2_OH), 58.5 (CO_2_C*C*H_2_O), 64.6 (CH_2_*C*CO_2_), 67.4 (CO_2_*C*H_2_CH_2_OH), 126.0, 128.1 (*C*_6_H_5_), 169.2 (CH_2_C*C*O_2_), 172.3 (*C*O_2_CHCl).

**ATRP Graft Copolymerization of MMA**

In a typical procedure, PHECPMA **2** (14.2 mg, *M*_n,GPC/MALS_ = 8,223 g/mol, *M*_w_/*M*_n_ = 1.08, *N*_HECPMA_ = 34.0, 0.06 mmol (-OCOCH(CH_3_)Cl group), CuCl (6.0 mg, 0.06 mmol), and dHbpy (21.2 mg, 0.06 mmol) were first added to a 10 mL Schlenk flask (flame-dried under vacuum prior to use) sealed with a rubber septum for degassing and kept under N_2_. Next, freshly-distilled DMF (1.92 mL), 2-butanone (1.92 mL), and MMA (3.85 mL, 36 mmol) were introduced via a gastight syringe. The solution was degassed by three cycles of freezing-pumping-thawing followed by stirring 5 min at room temperature. Finally, the flask was immersed into an oil bath set at 60^o^C. The polymerization lasted 20 min and was terminated by immersing the flask into liquid N_2_. The reaction mixture was diluted by THF and passed through an alumina column to remove the residual copper catalyst. The solution was concentrated and precipitated into diethyl ether. After repeated purification by dissolving in THF and precipitating in diethyl ether, 306 mg of white solid, PHEA-*g*-PMMA **3c**, was obtained after drying *in vacuo* overnight. GPC: *M*_n_ = 77,500 g/mol, *M*_w_/*M*_n_ = 1.34. FT-IR: *ν* (cm^-1^): 3438 (*ν*_O-H_), 2996, 2950, 1730 (*ν*_C=O_), 1487, 1449, 1387, 1361, 1274, 1241, 1192, 1149, 1061, 987, 911, 845, 749. ^1^H NMR (CDCl_3_): *δ* (ppm): 0.88, 1.03, 1.22 (3H, CH_2_C(C*H*_3_)CO_2_), 1.44, 1.82 (2H, C*H*_2_C(CH_3_)CO_2_), 1.90 (2H, *CH*_2_C(CH_2_O)CO_2_), 3.61 (3H, CO_2_*CH*_3_ and 2H, CO_2_CH_2_*CH*_2_OH), 3.95 (2H, CO_2_C*H*_2_CH_2_OH and 2H, CO_2_CC*H*_2_O).

**Determination of Critical Micelle Concentration**

PNA was used as fluorescence probe to measure the *cmc* of PHEA-*g*-PMMA **3** graft copolymer in aqueous media. Acetone solution of PNA ([PNA] = 2 mM) was added to a large amount of water until [PNA] reached 0.002 mM. The solutions for fluorescence measurement were obtained by adding different amounts of THF solutions of copolymer **3** (1, 0.1, 0.01, 0.001, or 0.0001 mg/mL) to water containing PNA ([PNA] = 0.002 mM).

**Micellar Morphology**

PHEA-*g*-PMMA **3** graft copolymer was added dropwise to deionized water under vigorous stirring until the concentration of copolymer **3** reached 0.1 mg/mL. THF was evaporated by stirring moderately overnight at room temperature. For TEM studies, 10 μL of micellar solution was deposited on an electron microscopy copper grid coated with carbon film and the water was evaporated at room temperature.

**Encapsulation of Hydrophobic Pyrene in Micelles**

THF solution containing PHEA-*g*-PMMA **3** graft copolymer and pyrene was added dropwise to deionized water under vigorous stirring until the concentration of copolymer **3** and pyrene reached 0.08 mg/mL and 0.01 mmol/L, respectively. THF was evaporated by stirring moderately overnight at room temperature. For the control experiment, only pyrene was added to deionized water. Both solutions were filtered through a 0.45 μm syringe ﬁlter and the obtained solutions were employed for the measurement of UV absorption spectroscopy.

**Encapsulation of Hydrophilic Rhodamine 6G in Micelles**

THF solution of PHEA-*g*-PMMA **3** graft copolymer was added dropwise to deionized water under vigorous stirring until the concentration of copolymer **3** reached 0.08 mg/mL. Next, aqueous solution of R6G was added to the above solution until the concentration of R6G reached 0.02 mmol/L. THF was evaporated by stirring moderately overnight at room temperature. The resulting solution was dialyzed against water using dialysis membrane (MW_cut-off_ = 3.5 kDa) until the dialysate did not show any detectable UV signal. The obtained solution was used for the measurements of UV absorption and fluorescence spectroscopy. Similar procedure was used for PEG_113_-*b*-PS_100_ diblock copolymer.

**Coencapsulation of Rhodamine 6G and Pyrene in Micelles**

THF solution containing PHEA-*g*-PMMA **3** graft copolymer and pyrene was added dropwise to deionized water under vigorous stirring until the concentration of copolymer **3** and pyrene reached 0.08 mg/mL and 0.01 mmol/L, respectively. R6G aqueous solution was then added to the above solution until the concentration of R6G reached 0.02 mmol/L. THF was evaporated by stirring moderately overnight at room temperature. The resulting solution was dialyzed against water using dialysis membrane (MW_cut-off_ = 3.5 kDa) until the dialysate did not show any detectable UV signal. The obtained solution was used for the measurement of UV absorption spectroscopy.

**References and Notes**

1. Jiang, X. Y.; Lu, G. L.; Feng, C.; Huang, X. Y. *Polym. Chem.* **2014**, *5*, 4915-4925.
2. Moad, G.; Chiefari, J.; Chong, Y. K.; Krstina, J.; Mayadunne, R. T. A.; Postma, A.; Rizzardo, E.; Thang, S. H. *Polym. Int.* **2000**, *49*, 993-1001.
3. Leduc, M. R.; Hawker, C. I.; Dao, J.; Frechet, J. M. J. *J. Am. Chem. Soc.* **1996**, *118*, 11111-11118.


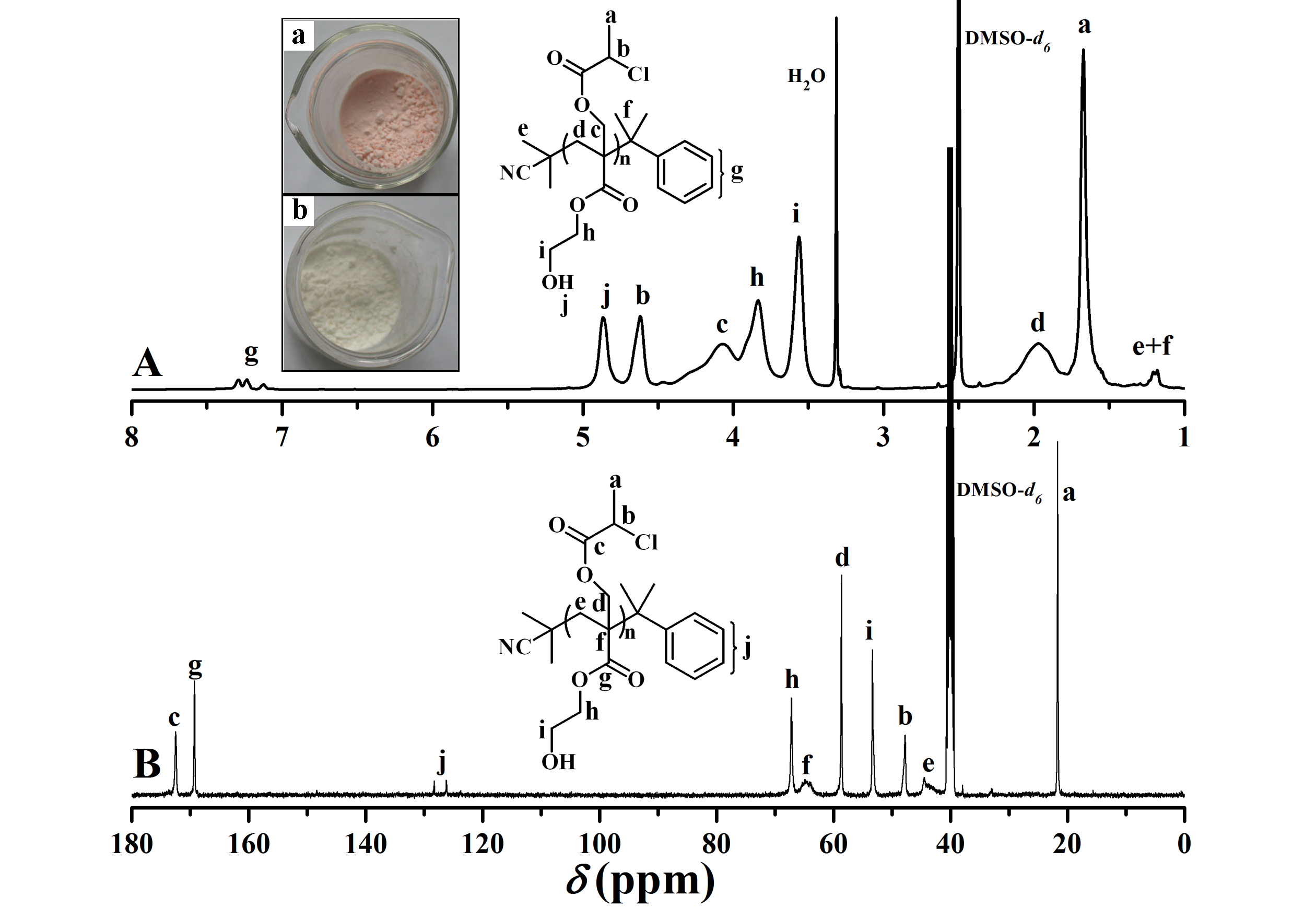


**Figure S1**. ^1^H (A) and ^13^C (B) NMR spectra of PHECPMA **2** in DMSO-*d*_6_.
